# Multimodal Tandem Mass Spectrometry Techniques for the Analysis of Phosphopeptides

**DOI:** 10.1021/jasms.1c00353

**Published:** 2022-05-23

**Authors:** Johanna Paris, Alina Theisen, Bryan P. Marzullo, Anisha Haris, Tomos E. Morgan, Mark P. Barrow, John O’Hara, Peter B. O’Connor

**Affiliations:** † Department of Chemistry, University of Warwick, Coventry CV4 7AL, United Kingdom; ‡ UCB, 216 Bath Road, Slough SL1 3WE, United Kingdom

**Keywords:** phosphopeptide, infrared multiphoton dissociation, ultraviolet photodissociation, electron capture dissociation, electron detachment dissociation, fragmentation, characterization, collision activated dissociation

## Abstract

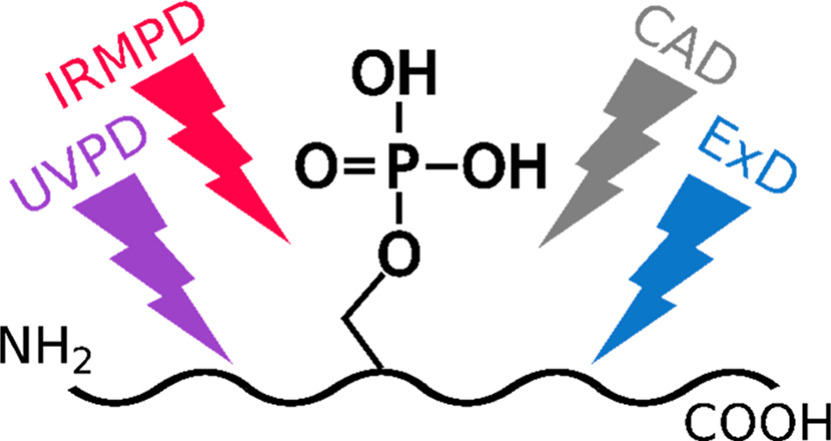

Collisionally activated dissociation (CAD), infrared multiphoton dissociation (IRMPD), ultraviolet photodissociation (UVPD), electron capture dissociation and electron detachment dissociation (EDD) experiments were conducted on a set of phosphopeptides, in a Fourier transform ion cyclotron resonance mass spectrometer. The fragmentation patterns were compared and varied according to the fragmentation mechanisms and the composition of the peptides. CAD and IRMPD produced similar fragmentation profiles of the phosphopeptides, while UVPD produced a large number of complementary fragments. Electron-based dissociation techniques displayed lower fragmentation efficiencies, despite retaining the labile phosphate group, and drastically different fragmentation profiles. EDD produced complex spectra whose interpretation proved challenging.

## Introduction

Phosphorylation is a posttranslational modification that occurs predominantly on serine, threonine, and tyrosine amino acid residues. Noncanonical phosphorylation may occur on other residues such as histidine, lysine, and arginine through phosphoamidate bonds and on aspartic acid and glutamic acid through anhydride linkages.^1−5^ The phosphoryl group is a labile modification that can be lost during mass spectrometry experiments, either by the hydrolysis of the phosphoramidate group by acidic additives frequently added to aid protonation of the analyte molecules during electrospray or by the dissociation technique itself. Loss of phosphoric acid (H_3_PO_4_) or metaphosphoric acid (HPO_3_) can be observed in the positive mode and loss of phosphite (PO_3_) in the negative mode. When the loss is induced by the fragmentation techniques, the phosphopeptides can be identified by immonium ions^6−8^ or the neutral losses can be used as a signature.^9−11^ Other strategies also allow the identification of phosphopeptides by accurate mass alone, using the mass defect of the phosphorus-31 isotope, ^31^P (−0.0262 Da),^12^ with the specific detection of ^31^P by ICP-MS,^13^ or by tagging the phosphate groups.^14^ However, the loss of the phosphate from the sequence ions makes phosphorylation site assignment difficult. The loss depends on the fragmentation techniques, the charge states of the precursor, the residue the phosphate is linked to, and the neighboring amino acids.^15,16^

During collisionally activated dissociation (CAD), the ions undergo multiple collisions with inert gas atoms such as argon, increasing the internal energy of the precursor ion until a threshold for dissociation is reached.^17^ The fragmentation occurs via a low energy proton rearrangement resulting in the destabilization and cleavage of an amide bond (b and y type fragments). The phosphoryl group is favored as a site for protonation, resulting in the cleavage of the labile group and a domination of the spectra by the loss of phosphoric acid (H_3_PO_4_). For in-beam CAD in a collision cell, such as CAD in a triple quadrupole or an FT-ICR instrument or higher-energy collisional dissociation (HCD),^7,18^ the collisions are more energetic than resonant-excitation CAD used in radio frequency quadrupole ion traps,^19^ resulting in greater intensities of sequence-informative b, y ions, compared to the phosphate neutral loss intensities. Evidence of rearrangement reactions in the gas phase has been exhibited in a broad range of peptides, under resonant CAD conditions.^20^ Furthermore, rearrangement of the phosphoryl group to an alternate hydroxyl-containing peptide has also been observed.^19^ The time scale of the activation could also allow the transfer of the phosphate group to another residue in resonant CAD experiments.^15^

Electron-based fragmentation techniques are radical-driven dissociation methods where the labile groups are often retained,^21−23^ allowing identification and localization of the phosphorylation. Electron-capture dissociation (ECD) involves the capture of a low-energy electron by a multiply charged precursor cation.^24^ Fragmentation of the N–Cα bond of the peptide backbone produces predominantly c- and z-type product ions. Electron-detachment dissociation (EDD) consists of the use of electrons with kinetic energies above 10 eV to detach electrons from a negatively charged precursor in negative-mode analysis.^25^ The negative charges are located at the amide nitrogen bond along the backbone, producing a^•^/x fragments, or at the amino acid side chain, producing neutral losses.^26,27^ Fragmentation efficiency and sequence coverage with electron-based techniques are lower when a peptide is phosphorylated.^28,29^ On phosphopeptides with basic residues, the phosphate group can exist in a deprotonated form and can form salt bridges with protonated side chains.^30,31^ Salt bridges are electrostatic interactions between amino acids of different charges.^32^ These strong noncovalent bonds stabilize the phosphopeptide ion, and additional electron energy is therefore necessary to dissociate it.^33−35^ Basic residues are common around phosphorylation sites, suggesting that salt bridges could be part of their formation.^36^

Different lasers can be used to dissociate ions. In infrared multiphoton dissociation (IRMPD),^37−39^ ions are irradiated using a CO_2_ laser. The sequential absorption of 10.6 μm photons increases the internal energy of ions (0.117 eV per photon) until dissociation occurs. It is a slow heating method, similar to CAD, and the energy is distributed throughout the peptide, resulting in bond cleavage at the weakest points (b and y fragments, labile posttranslational modifications).^40,41^ Infrared (IR) radiation is resonant with the phosphate vibrational modes of the phosphorylated peptide,^42,43^ leading to enhanced fragmentation of phosphopeptides.^44−47^ Ultraviolet photodissociation (UVPD) at 193 nm deposits the required energy for dissociation within the absorption of a single photon^48^ (6.4 eV per photon) emitted through a nanosecond-scale laser pulse.^49^ The peptide absorbs the high-energy photon at the chromophore and the protein backbone amide group,^50^ accessing many dissociation pathways, leading to the generation of a/x, b/y, and c/z complementary ion pairs.^51,52^ The fast fragmentation of the peptide reduces the frequency of phosphate group loss.^53^

The 12 T solariX FT-ICR mass spectrometer is a versatile instrument. CAD fragmentation is possible in the front end. The addition of lasers allows fragmentation via IRMPD and UVPD in the ICR cell.^54,55^ Finally, the hollow beam electrode allows ExD fragmentation. CAD, IRMPD, UVPD, ECD, and EDD fragmentation of a phosphoserine, a phosphothreonine, and a phosphotyrosine peptide were conducted and compared in this paper.

## Methods

MS Phosphomix 1 and 2 lights were obtained from Sigma-Aldrich (MSP1L-1VL and MSP2L-1VL). Water was purified by a Millipore Direct-Q purification system (Merck Millipore, MA). Acetonitrile was obtained from VWR chemicals (CAS: 75-05-8). Formic acid (FA) was obtained from Sigma-Aldrich (CAS: 64-18-6). The Phosphomix were diluted into 80:20 water/ACN + 0.1% FA, and the final concentration of the peptides was 0.2 μM.

The samples were ionized using a custom nano electrospray ionization source (nESI). A 10–20 μL portion of sample was loaded into a pulled glass capillary tip with a several micrometer open orifice^56^ and analyzed with a 12 T Bruker solariX FTICR mass spectrometer (Bruker Daltonik GmbH, Bremen, Germany). The applied capillary voltage was 500 V.

For CAD, argon was used as the neutral collision gas (∼7 × 10^–6^ mbar). Ions were isolated in the quadrupole, accumulated, and fragmented in the collision cell with an optimized collision potential and then analyzed in the infinity cell.^55^

For ECD, IRMPD, and UVPD, the peptides were isolated in the quadrupole, accumulated in the collision cell, and transferred into the ICR cell where the ions were fragmented, excited, and detected.

For ECD, ions were irradiated with electrons from a 1.5 A indirectly heated hollow cathode by applying a bias voltage of 1.2 V. Below 1.2 V, no fragmentation was observed. Various pulse lengths were applied, and the best spectra are shown in the figures. Higher and lower pulse lengths than the optimized ones occurred, and the lower number of sequence ions is shown. IRMPD fragmentation was achieved using a continuous wave, 25 W, CO_2_ laser (Synrad Inc., Mulkiteo, WA). UVPD was performed using a 193 nm excimer laser (ExciStar XS Coherent, 500 Hz). Ions were irradiated with a varying number of shots with a pulse energy of 5 mJ (measured at laser output).

RDSLGpTYSSR was quadrupole isolated at *m*/*z* 612.5 ± 4, and the acquired mass-to-charge ratio range was *m*/*z* 147.4–3000. EVQAEQPSSpSSPR was quadrupole isolated at *m*/*z* 741.0 ± 4, and the acquired mass-to-charge ratio range was *m*/*z* 98.3–3000. VIEDNEpYTAR was quadrupole isolated at *m*/*z* 647.0 ± 10, and the acquired mass-to-charge ratio range was *m*/*z* 98.3–1300. The mass spectrometer was tuned to get the best signal for each spectrum, and data was acquired at different fragmentation parameters. The spectra with the best fragmentation efficiency, with a high number of sequence ions, low internal fragmentation, and phosphate loss, are shown in this paper and compared. Data points (4 M, 2,^22^ 22-bit) were acquired for each spectrum. For CAD, IRMPD, and UVPD 100 scans were averaged, for ECD 200 scans, and for EDD 500 scans to achieve a desirable signal-to-noise ratio. The data was internally calibrated using known fragment peaks with a quadratic calibration function in the Bruker DataAnalysis v4.3 software (Bruker Daltonics GmbH, Bremen, Germany).

## Results

**Figure 1:**
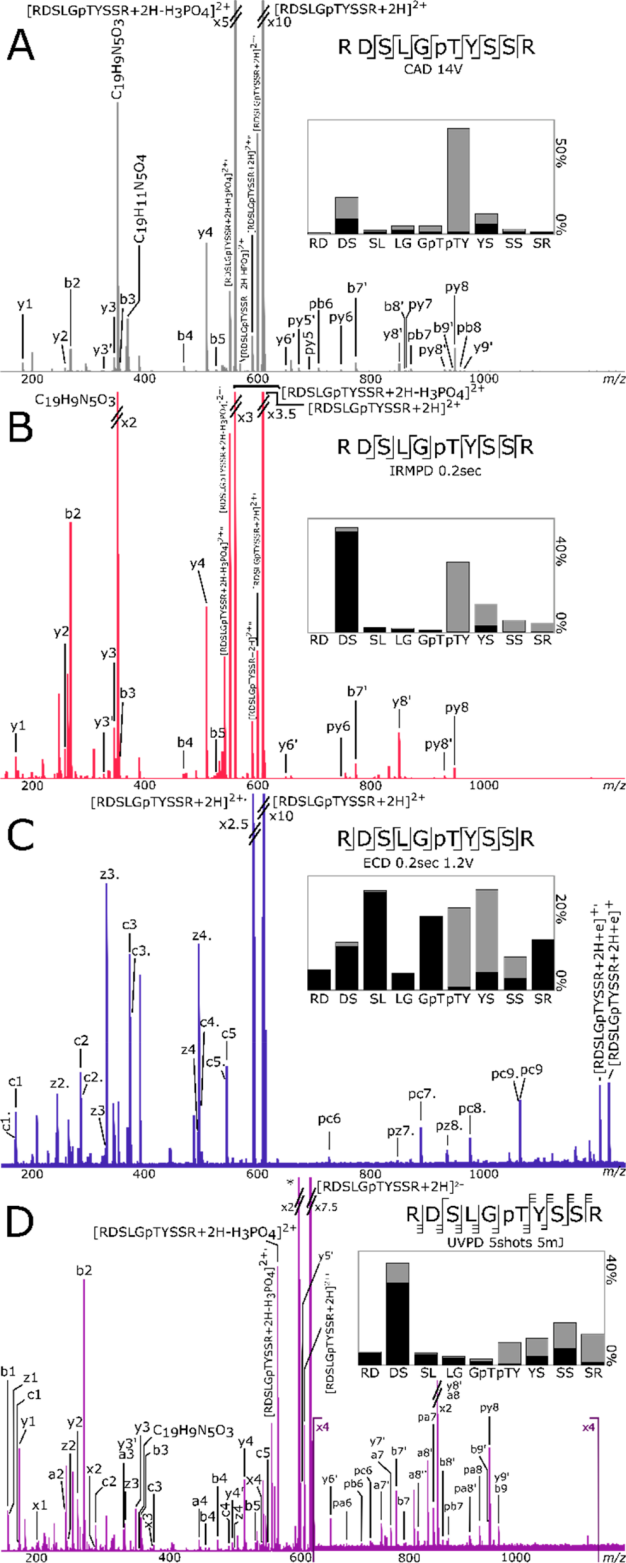
(A) CAD, (B) IRMPD, (C) ECD, and (D) UVPD fragmentation of RDSLGpTYSSR. p: fragment with phosphate attached. ′: loss of water. ′′: loss of two water molecules. Only fragments without neutral losses are shown on the cleavage diagrams. On the fragmentation intensity profiles, black and gray represent the summed percentages of abc and xyz, respectively.

Fragmentation spectra of doubly charged RDSLGpTYSSR are shown in Figure 1. CAD and IRMPD spectra show high similarities and have similar fragmentation profiles. Both fragmentation techniques localized the phosphorylation site at the threonine. The highest intensity sequence ions were b2 for IRMPD and y4 for both fragmentation techniques, demonstrating preferential cleavage at DS and pTY linkages. RDSLGpTYSSR is composed of two basic arginine residues, which sequestered the two ionizing protons. Therefore, no mobile proton and no charge delocalization occurred, making the charge-remote dissociation channels competitive. The peptide dissociates selectively at the C-terminus of the two acidic residues (aspartic acid and phosphothreonine) as shown in previous fragmentation studies.^41,57,58^

Cleavage between the R and D residues was not observed. Neutral loss of H_3_PO_4_ was prominent with CAD and IRMPD (27.4% and 83.7% respectively, in the [M + 2H – H_3_PO_4_]^2+^/[M + 2H]^2+^ ratio), and CAD also produced loss of HPO_3_ (0.2% [M + 2H – HPO_3_]^2+^/[M + 2H]^2+^). Fragments with water losses (′ refers to loss of water) were observed: y5–9′, b7–9′ for CAD and y5–7′ and b7′ for IRMPD, representing 10.0% and 3.8%, respectively, of the identified sequence ion intensities. However, the fragments have their phosphorylated counterpart, and the phosphorylation site is easily identified on the threonine. Both fragmentation techniques produced internal fragments identified as C_19_H_9_N_5_O_3_^+^ for CAD and IRMPD (∼−0.17 ppm for both) and C_19_H_11_N_5_O_4_^+^ for CAD (−0.07 ppm). The percentage of internal fragments and rearrangements (the percentage of unknown peaks and internal fragments compared to the total ion intensity) was 3.5% for CAD and 25.6% for IRMPD with the prominence of C_19_H_9_N_5_O_3_ in the IRMPD spectrum.

For peptides with no mobile proton, it has been shown that the hydrogen bond between the phosphate group and the protonated arginine residue can lead to the gas-phase rearrangement of the phosphate to a different residue.^19^ However, no ions resulting from such rearrangement were observed in the spectra (y2, y3, and y4 with phosphate).

UVPD produced a large number of complementary fragments, leading to 100% cleavage coverage. The fragmentation profile shows high intensity fragments originating from the DS linkage. UVPD did not dissociate selectively at the pTY linkage compared to the slow heating techniques.

UVPD produced an internal fragment assigned as C_19_H_9_N_5_O_3_ (−0.17 ppm), and 28.3% of the intensity of the peaks observed were due to internal fragmentation (the high percentage is due to the unidentified high intensity *m*/*z* 593.15759). UVPD lost less of the phosphate group from the precursor (9.0%) than the slow heating methods, but more of the identified fragments were dephosphorylated (17.5%). However, the phosphorylated sequence ions allowed the identification of the phosphorylated site at the threonine.

The ECD fragmentation profiles show more homogeneous fragmentation through the backbone of the peptide, leading to 100% cleavage coverage. No selective cleavage was observed compared to the other techniques. There was no loss of phosphate from the precursor or the fragments, permitting the identification of the phosphorylation site at the threonine. Starting from a similar precursor intensity, prior to fragmentation, ECD required the accumulation of more scans than the other fragmentation techniques due to fragment peaks being observed with lower intensities (between ∼2 to 5 times lower). This phenomenon is expected with ECD fragmentation but is also possibly enhanced by the phosphorylation.

**Figure 2:**
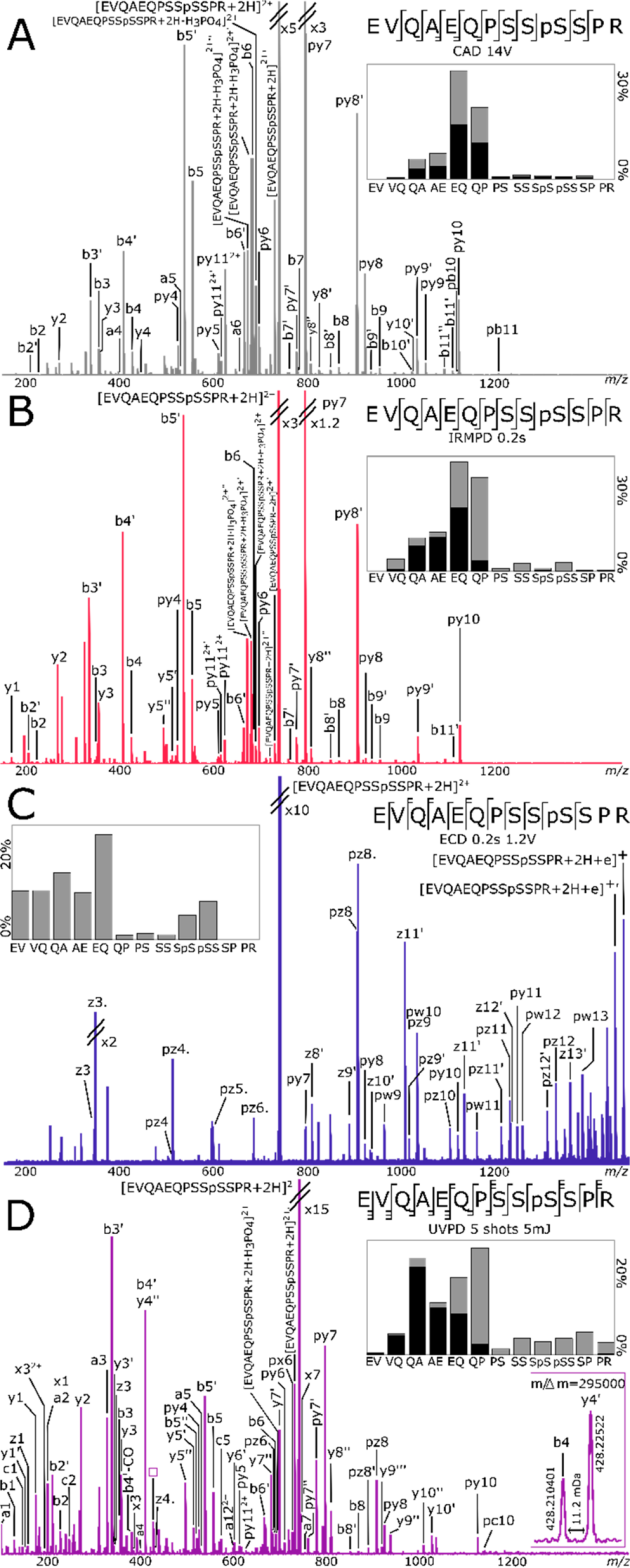
(A) CAD, (B) IRMPD, (C) ECD, and (D) UVPD fragmentation of EVQAEQPSSpSSPR. p: fragment with phosphate attached. ′: loss of water. ′′: loss of two water molecules. Only fragments without neutral losses are shown on the cleavage diagrams. On the fragmentation intensity profiles, black and gray represent the summed percentages of abc and xyz, respectively.

Fragmentation spectra of the doubly charged EVQAEQPSSpSSPR are shown in Figure 2. CAD and IRMPD techniques produced similar fragmentation profiles, with high abundance complementary fragments arising from the EQ (py8/b5) and QP (py7/b6) cleavages. The glutamic acid residue has been shown to produce similar selective fragmentation to asparagine.^59^ With slow heating methods, the high gas-phase basicity of the proline compared to the other residues with alkyl side chains enhances the dissociation of the amide bond at the N-terminus side.^60^ As expected, the dominant fragment ion in the CAD and IRMPD spectra was the y fragment between the Q and P residues (denoted py7 because it is a phosphorylated y7 ion).

The slow heating techniques produced limited fragments at the QA and AE linkage and very low fragmentation with PSSpSSEPR. Fragment with EV cleavages were not identified. With both fragmentation techniques, H_3_PO_4_ losses were identified at the precursor, as well as loss of one, two, or three H_2_O. Fragments with phosphate losses are observed: y5–9′, b7–9′ for CAD and y5–7′, b7′ for IRMPD, representing 10.0% and 3.8%, respectively, of the identified sequence ion intensities. However, the fragments have their phosphorylated counterpart, and the phosphorylation site is easily identified on the serine.

UVPD resulted in fragmentation at every backbone linkage, with complementary fragments, leading to 100% cleavage coverage and the most complete fragmentation for this peptide. Most of the fragments have less than half of the mass of the precursor, charged at one of the extremities of the peptide. There was lower fragmentation at EV and PSSpSSPR.

The ECD fragmentation data only shows fragments from the C-terminus. The proton captured the electron, and the only charge left was the one sequestered at the C-terminus arginine.

The C-terminus (SPR) was not fragmented. Some z fragments were found with a side chain loss corresponding to C_3_H_5_NO (pw9,11–13) likely from the glutamine residues.^61^ Four y fragments were produced (py7–8,10–11). The percentage of unidentified internal fragments or rearrangements (14.7%) for ECD is similar to the percentages for CAD, IRMPD, and UVPD (15.6%, 25.6%, and 17.7%) and arise mostly from neutral losses of the electron capture species. Fragments with loss of the phosphate group were found (z8–12′) at unexpectedly higher percentage intensities (23.6%) than with CAD (4.5%), IRMPD (7.0%), and UVPD (19.3%). MS/MS of EVQAEQPSSpSSPR with ECD gave low-intensity fragments suggesting an inner stabilization of the peptide.

Despite its mobile proton, and the proline residue at the C-terminus of the peptide, the doubly charged EVQAEQPSSpSSPR has low fragmentation efficiency at PSSpSSPR around the site of the phosphorylation, in all spectra with all dissociation techniques. In addition, the neutral loss of H_3_PO_4_ from the precursor was low with all techniques (respectively 4.7%, 1.7%, 0%, and 0.4% for CAD, IRMPD, ECD, and UVPD in the [M + 2H – H_3_PO_4_]^2+^/[M + 2H]^2+^ ratio), compared to the loss in the two other model peptides, and there was no loss of HPO_3_ with any of the fragmentation techniques. These two observations, and the ECD fragmentation behavior, suggest an inner stabilization that could be due to a strong hydrogen-bonding interaction between the phosphoserine and the protonated arginine residue.^33,35^ The use of activated ion ECD, with the simultaneous irradiation of the IR laser, could potentially separate the ECD ions which are held together by a hydrogen bond, testing this hypothesis.^54,62^

**Figure 3:**
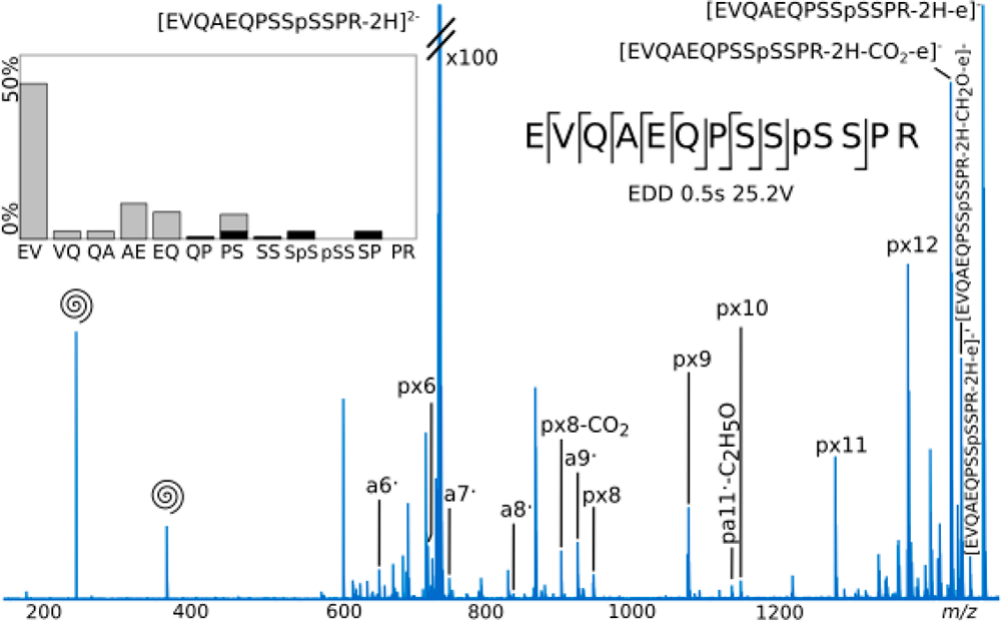
EDD fragmentation of EVQAEQPSSpSSPR. p: fragment with phosphate attached. ′: loss of water. Swirl: harmonics. Only fragments without neutral losses are shown on the cleavage diagrams. On the fragmentation intensity profiles, black and gray represent the summed percentages of abc and xyz, respectively.

EVQAEQPSSpSSPR was fragmented in the negative mode via electron-detachment dissociation (EDD). Low intensity a^•^ and x fragments were observed in the spectra (Figure 3), as well as neutral losses (CO_2_, C_2_H_5_O, CH_2_O). The labile phosphate was not cleaved from the precursor or the fragments. Despite the low fragmentation efficiency, the ions gave sequence information localizing the phosphorylation between the third and fourth serine.

The fragmentation profile shows a high intensity fragment arising from the EV cleavage (px12). This data is consistent with the electron–hole recombination phenomenon,^25^ where the loss of the electron produces a radical with a positive charge, mobile via Coulombic attraction on the backbone, and neutralized at a negative amino acid such as the glutamic acid (E).The tuning for EDD was more challenging than for the other fragmentation techniques. With CAD, IRMPD, ECD, and UVPD, most of the acquired spectra showed the same product ions and fragmentation profiles, with different results when reaching extreme parameters. For EDD, optimal data was obtained for EVQAEQPSSpSSPR using a bias voltage of 25.2 V and pulse length of 0.5 s. Some fragments were not observed at 25 and 25.4 V bias or at 25.2 V with pulse lengths of 0.3 or 0.4 s. This narrow tuning window demonstrates the difficulty of the fragmentation tuning but also the complexity of the EDD mechanism.^63^ EDD required more averaged scans to get good signal-to-noise ratio (500 scans). Furthermore, the fragmentation of RDSLGpTYSSR and VIEDNEpYTAR led to complex spectra with mostly unidentified fragments. Only px9, px8, and px7 were identified for RDSLGpTYSSR (data not shown).

**Figure 4:**
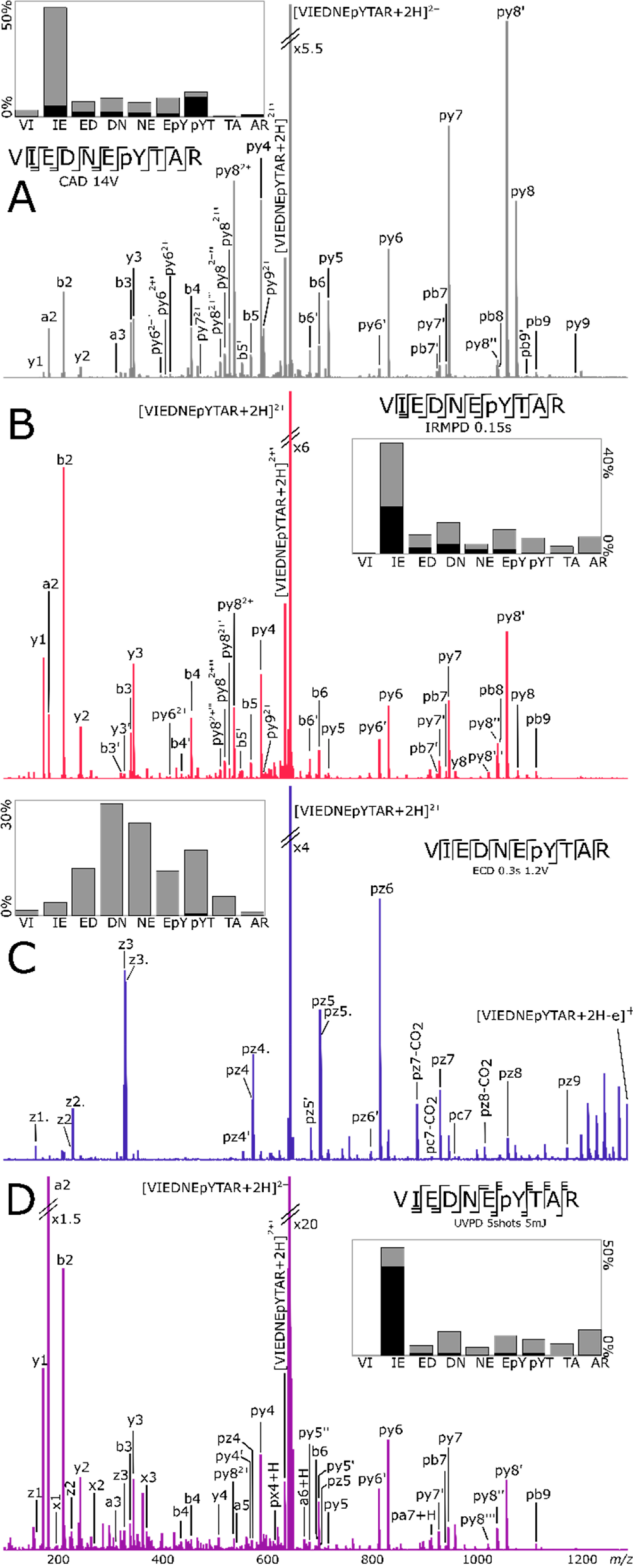
(A) CAD, (B) IRMPD, (C) ECD, and (D) UVPD fragmentation of VIEDNEpYTAR. p: fragment with phosphate attached. ′: loss of water. ′′: loss of two water molecules. Only fragments without neutral losses are shown on the cleavage diagrams On the fragmentation intensity profiles, black and gray represent the summed percentages of abc and xyz, respectively. All four spectra are on the same horizontal scale.

Fragmentation spectra of doubly charged VIEDNEpYTAR are shown in Figure 4.CAD, IRMPD and UVPD displayed similar fragmentation profiles with selective dissociation at the IE linkage. a2, b2, py8, and/or py8′ fragments were detected at high intensities in the three spectra. The dominant fragment ions are observed to arise from cleavage reactions at the N-terminus of the glutamic acid. The peptide contains three acidic residues (2xE and D), and surprisingly, the selective fragmentation was detected at the glutamic acid residue only.

The C-terminus arginine sequestered one of the protons and the electron was captured by the proton, resulting in most of the ECD fragments containing the C-terminus. The central part of the peptide, where all the acidic residues were located, was more prone to fragmentation than the extremities of the peptide. Neutral losses from the electron-capture species such as CO_2_

Phosphotyrosine cannot undergo the direct loss of H_3_PO_4_ or HPO_3_ from the precursor, via β-elimination reaction. VIEDNEpYTAR spectra (Figure 4) displayed no loss of the phosphate group from the precursor, and there is no fragment suggesting the transfer of the phosphate. CAD and ECD spectra contain no fragment that would indicate a loss of phosphate. Laser fragmentation techniques produced low-intensity fragments with a loss of phosphate: 0.4% for IRMPD and 2.2% for UVPD of the intensity of the identified fragments y4, y4′, y6′, y6″, and y8′ for IRMPD and y4, y4′, y6, and y6′ for UVPD, which is lower than for the other peptides. Phosphotyrosine is less common than phosphoserine and phosphothreonine.^15^

The percentages of internal fragments or rearrangements are 3.1%, 5.7%, and 11.5% for CAD, IRMPD, and UVPD, which are lower than for RDSLGpTYSSR and EVQAEQPSSpSSPR. RDSLGpTYSSR showed the higher internal fragmentation percentages due to the high intensities of its two main internal fragments, and EVQAEQPSSpSSPR showed higher neutral and side-chain losses than the two other peptides. CAD, IRMPD, and ECD produced 100% cleavage coverage, while the UVPD did not cleave at the VI linkage.

## Discussion

FT-ICR mass spectrometers are versatile instruments where multiple fragmentation techniques can be implemented and compared. For the characterization of the three selected phosphopeptides, the two slow heating methods (CAD and IRMPD) produced similar fragmentation profiles with very little variation between the spectra. With the IRMPD technique, the fragments remain in the laser beam during the irradiation time and can be further fragmented; therefore, higher percentages of internal fragments are detected in the IRMPD spectra than the CAD spectra of the three peptides. These two fragmentation techniques can be distinguished by different characteristics. First, in the design of the instrument, the CAD fragmentation occurs in the collision cell at the front end of the mass spectrometer, and the IRMPD fragmentation occurs in the ICR cell. Second, it has been shown that the phosphopeptide fragment via IRMPD dissociation occurs at a much lower threshold than its unphosphorylated counterparts.^46,47^ These two characteristics of IRMPD fragmentation compared to CAD can be useful; for example, IRMPD can be used to perform a two-dimensional mass spectrometry (2DMS) analysis of a phosphoproteomic sample.^11^ It is also possible to perform sustained off-resonance irradiation collisionally activated dissociation (SORI-CAD) in the ICR cell; however, in-cell fragmentation requires an increase in pressure and therefore reduces the resolving power of the experiment.

UVPD fragmentation produced a lot of complementary fragments. No significant variation of fragmentation efficiency was detected between the phosphoserine, phosphothreonine, and phosphotyrosine peptides. Selective fragmentation at the N-terminus of proline and around the acidic amino acid residues was observed in the CAD, IRMPD, and UVPD spectra, especially when the protons were immobilized. In these cases, competing fragmentation channels are discriminated against. Selective cleavages were observed with UVPD in peptides 1 and 3. A high-energy photon can cause dissociation via three pathways: the absorption leads to an electron shifting to an electronic excited state, breaking a bond into two radicals, one hot, one cold. The hot radical is beyond the threshold of dissociation resulting in direct cleavage. The cold radical undergoes radical rearrangements similar to ECD, and internal conversion of the electronic energy to vibrational modes results in even-electron fragmentation similar to CAD. These latter even-electron fragmentations lead to selective cleavages The phosphotyrosine peptide had a UVPD fragmentation profile similar to that of the slow-heating methods such as CAD and IRMPD, while the UVPD fragmentation profiles of the phosphoserine and phosphothreonine peptides had patterns of all of the ECD, CAD, and IRMPD profiles. This difference is likely due to the nature of the phosphorylated amino acid, which is a UV chromophore. The use of electron-based fragmentation techniques did not show selective dissociation, as expected, but was less effective in the fragmentation of the phosphopeptides. Despite the more homogeneous fragmentation profile, and the ability to retain the labile phosphorylation, ECD produced lower intensity fragments, and rearrangements, due to an inner stabilization caused by the phosphorylation. EDD fragmentation in the negative mode produced unidentified fragments and difficult spectra to interpret, leading to low sequence information.

CAD, IRMPD, ECD, and UVPD produced complementary sequence information and permitted the localization of the phosphate group. No phosphate transfer between the phosphoamino acid residue and another amino acid residue of the peptide were detected. The cleavage coverage was around 100% for all spectra with exceptions relating to the composition of the peptides more than the ability of the fragmentation techniques.

## Supporting Information Available

Tables of peak assignments of the spectra (PDF)

## References

[ref1] Zu X.-L.; Besant P.; Imhof A.; Attwood P. Mass spectrometric analysis of protein histidine phosphorylation. Amino acids 2007, 32, 347–357. 10.1007/s00726-007-0493-4. 17334905

[ref2] Attwood P.; Piggott M.; Zu X.; Besant P. Focus on phosphohistidine. Amino acids 2007, 32, 145–156. 10.1007/s00726-006-0443-6. 17103118

[ref3] Kleinnijenhuis A. J.; Kjeldsen F.; Kallipolitis B.; Haselmann K. F.; Jensen O. N. Analysis of Histidine Phosphorylation Using Tandem MS and Ion– Electron Reactions. Anal. Chem. 2007, 79, 7450–7456. 10.1021/ac0707838. 17822303

[ref4] Cieśla J.; Frączyk T.; Rode W. Phosphorylation of basic amino acid residues in proteins: important but easily missed. Acta Biochim. Polym. 2011, 58, 137–148. 10.18388/abp.2011_2258. 21623415

[ref5] Hardman G.; Perkins S.; Brownridge P. J.; Clarke C. J.; Byrne D. P.; Campbell A. E.; Kalyuzhnyy A.; Myall A.; Eyers P. A.; Jones A. R. Strong anion exchange-mediated phosphoproteomics reveals extensive human non-canonical phosphorylation. EMBO J. 2019, 38, e100847 10.15252/embj.2018100847. 31433507 PMC6826212

[ref6] Wilm M.; Neubauer G.; Mann M. Parent ion scans of unseparated peptide mixtures. Anal. Chem. 1996, 68, 527–533. 10.1021/ac950875+. 8712361

[ref7] Olsen J. V.; Macek B.; Lange O.; Makarov A.; Horning S.; Mann M. Higher-energy C-trap dissociation for peptide modification analysis. Nat. Methods 2007, 4, 709–712. 10.1038/nmeth1060. 17721543

[ref8] Huddleston M. J.; Annan R. S.; Bean M. F.; Carr S. A. Selective detection of phosphopeptides in complex mixtures by electrospray liquid chromatography/mass spectrometry. JASMS 1993, 4, 710–717. 10.1016/1044-0305(93)80049-5. 24225996

[ref9] Qin J.; Chait B. T. Identification and characterization of posttranslational modifications of proteins by MALDI ion trap mass spectrometry. Anal. Chem. 1997, 69, 4002–4009. 10.1021/ac970489n. 9322437

[ref10] Schlosser A.; Pipkorn R.; Bossemeyer D.; Lehmann W. D. Analysis of protein phosphorylation by a combination of elastase digestion and neutral loss tandem mass spectrometry. Anal. Chem. 2001, 73, 170–176. 10.1021/ac000826j. 11199962

[ref11] Paris J.; Morgan T. E.; Wootton C. A.; Barrow M. P.; O’Hara J.; O’Connor P. B. Facile determination of phosphorylation sites in peptides using two-dimensional mass spectrometry. Anal. Chem. 2020, 92, 6817 10.1021/acs.analchem.0c00884. 32286050

[ref12] Mao Y.; Zamdborg L.; Kelleher N. L.; Hendrickson C. L.; Marshall A. G. Identification of phosphorylated human peptides by accurate mass measurement alone. Int. J. Mass spectrom. 2011, 308, 357–361. 10.1016/j.ijms.2011.08.006. 22866021 PMC3409838

[ref13] Wind M.; Wesch H.; Lehmann W. D. Protein phosphorylation degree: determination by capillary liquid chromatography and inductively coupled plasma mass spectrometry. Anal. Chem. 2001, 73, 3006–3010. 10.1021/ac010066s. 11467547

[ref14] Chen M.; Su X.; Yang J.; Jenkins C. M.; Cedars A. M.; Gross R. W. Facile identification and quantitation of protein phosphorylation via β-elimination and Michael addition with natural abundance and stable isotope labeled thiocholine. Anal. Chem. 2010, 82, 163–171. 10.1021/ac9015193. 20000356 PMC2813211

[ref15] DeGnore J. P.; Qin J. Fragmentation of phosphopeptides in an ion trap mass spectrometer. JASMS 1998, 9, 1175–1188. 10.1016/S1044-0305(98)00088-9. 9794085

[ref16] Rožman M. Modelling of the gas-phase phosphate group loss and rearrangement in phosphorylated peptides. J. Mass Spectrom. 2011, 46, 949–955. 10.1002/jms.1974. 21915960

[ref17] Wysocki V. H. Internal energy effects in mass spectrometry; Purdue University, 1987.

[ref18] Zhang Y.; Ficarro S. B.; Li S.; Marto J. A. Optimized Orbitrap HCD for quantitative analysis of phosphopeptides. JASMS 2009, 20, 1425–1434. 10.1016/j.jasms.2009.03.019. 19403316

[ref19] Palumbo A. M.; Reid G. E. Evaluation of gas-phase rearrangement and competing fragmentation reactions on protein phosphorylation site assignment using collision induced dissociation-MS/MS and MS3. Anal. Chem. 2008, 80, 9735–9747. 10.1021/ac801768s. 19012417

[ref20] Mouls L.; Aubagnac J.-L.; Martinez J.; Enjalbal C. Low energy peptide fragmentations in an ESI-Q-Tof type mass spectrometer. J. Proteome Res. 2007, 6, 1378–1391. 10.1021/pr060574o. 17311442

[ref21] Stensballe A.; Jensen O. N.; Olsen J. V.; Haselmann K. F.; Zubarev R. A. Electron capture dissociation of singly and multiply phosphorylated peptides. Rapid Commun. Mass Spectrom. 2000, 14, 1793–1800. 10.1002/1097-0231(20001015)14:19<1793::AID-RCM95>3.0.CO;2-Q. 11006587

[ref22] Kowalewska K.; Stefanowicz P.; Ruman T.; Frączyk T.; Rode W.; Szewczuk Z. Electron capture dissociation mass spectrometric analysis of lysine-phosphorylated peptides. Biosci. Rep. 2010, 30, 433–443. 10.1042/BSR20090167. 20144148 PMC2947194

[ref23] Voinov V. G.; Bennett S. E.; Beckman J. S.; Barofsky D. F. ECD of tyrosine phosphorylation in a triple quadrupole mass spectrometer with a radio-frequency-free electromagnetostatic cell. JASMS 2014, 25, 1730–1738. 10.1007/s13361-014-0956-2. PMC4163116 25037842

[ref24] Zubarev R. A.; Kelleher N. L.; McLafferty F. W. Electron capture dissociation of multiply charged protein cations. A nonergodic process. J. Am. Chem. Soc. 1998, 120, 3265–3266. 10.1021/ja973478k.

[ref25] Budnik B. A.; Haselmann K. F.; Zubarev R. A. Electron detachment dissociation of peptide di-anions: an electron–hole recombination phenomenon. Chem. Phys. Lett. 2001, 342, 299–302. 10.1016/S0009-2614(01)00501-2.

[ref26] Anusiewicz I.; Jasionowski M.; Skurski P.; Simons J. Backbone and side-chain cleavages in electron detachment dissociation (EDD). J. Phys. Chem. A 2005, 109, 11332–11337. 10.1021/jp055018g. 16331920

[ref27] Kjeldsen F.; Silivra O. A.; Ivonin I. A.; Haselmann K. F.; Gorshkov M.; Zubarev R. A. Cα–C Backbone Fragmentation Dominates in Electron Detachment Dissociation of Gas-Phase Polypeptide Polyanions. Chem.—Eur. J. 2005, 11, 1803–1812. 10.1002/chem.200400806. 15672435

[ref28] Creese A. J.; Cooper H. J. The effect of phosphorylation on the electron capture dissociation of peptide ions. JASMS 2008, 19, 1263–1274. 10.1016/j.jasms.2008.05.015. PMC2570175 18585055

[ref29] Moss C. L.; Chung T. W.; Wyer J. A.; Nielsen S. B.; Hvelplund P.; Tureček F. Dipole-guided electron capture causes abnormal dissociations of phosphorylated pentapeptides. JASMS 2011, 22, 731–751. 10.1007/s13361-011-0083-2. 21472611

[ref30] Kim D.; Pai P.-J.; Creese A. J.; Jones A. W.; Russell D. H.; Cooper H. J. Probing the electron capture dissociation mass spectrometry of phosphopeptides with traveling wave ion mobility spectrometry and molecular dynamics simulations. JASMS 2015, 26, 1004–1013. 10.1007/s13361-015-1094-1. PMC4422852 25832028

[ref31] Woods A. S. The mighty arginine, the stable quaternary amines, the powerful aromatics, and the aggressive phosphate: their role in the noncovalent minuet. J. Proteome Res. 2004, 3, 478–484. 10.1021/pr034091l. 15253429

[ref32] Kumar S.; Nussinov R. Close-range electrostatic interactions in proteins. ChemBioChem. 2002, 3, 604–617. 10.1002/1439-7633(20020703)3:7<604::AID-CBIC604>3.0.CO;2-X. 12324994

[ref33] Jackson S. N.; Wang H.-Y. J.; Woods A. S. Study of the fragmentation patterns of the phosphate-arginine noncovalent bond. J. Proteome Res. 2005, 4, 2360–2363. 10.1021/pr050261d. 16335986

[ref34] Jackson S. N.; Wang H.-Y. J.; Yergey A.; Woods A. S. Phosphate stabilization of intermolecular interactions. J. Proteome Res. 2006, 5, 122–126. 10.1021/pr0503578. 16396502 PMC2538564

[ref35] Woods A. S.; Ferré S. Amazing stability of the arginine– phosphate electrostatic interaction. J. Proteome Res. 2005, 4, 1397–1402. 10.1021/pr050077s. 16083292 PMC2945258

[ref36] Iakoucheva L. M.; Radivojac P.; Brown C. J.; O’Connor T. R.; Sikes J. G.; Obradovic Z.; Dunker A. K. The importance of intrinsic disorder for protein phosphorylation. Nucleic Acids Res. 2004, 32, 1037–1049. 10.1093/nar/gkh253. 14960716 PMC373391

[ref37] Little D. P.; Speir J. P.; Senko M. W.; O’Connor P. B.; McLafferty F. W. Infrared multiphoton dissociation of large multiply charged ions for biomolecule sequencing. Anal. Chem. 1994, 66, 2809–2815. 10.1021/ac00090a004. 7526742

[ref38] Talebpour A.; Bandrauk A.; Yang J.; Chin S. Multiphoton ionization of inner-valence electrons and fragmentation of ethylene in an intense Ti: sapphire laser pulse. Chem. Phys. Lett. 1999, 313, 789–794. 10.1016/S0009-2614(99)01075-1.

[ref39] Maitre P.; Scuderi D.; Corinti D.; Chiavarino B.; Crestoni M. E.; Fornarini S. Applications of Infrared Multiple Photon Dissociation (IRMPD) to the Detection of Posttranslational Modifications. Chem. Rev. 2020, 120, 3261 10.1021/acs.chemrev.9b00395. 31809038

[ref40] McCormack A. L.; Somogyi A.; Dongre A. R.; Wysocki V. H. Fragmentation of protonated peptides: surface-induced dissociation in conjunction with a quantum mechanical approach. Anal. Chem. 1993, 65, 2859–2872. 10.1021/ac00068a024. 8250266

[ref41] Tsaprailis G.; Nair H.; Somogyi Á.; Wysocki V. H.; Zhong W.; Futrell J. H.; Summerfield S. G.; Gaskell S. J. Influence of secondary structure on the fragmentation of protonated peptides. J. Am. Chem. Soc. 1999, 121, 5142–5154. 10.1021/ja982980h.

[ref42] Correia C. F.; Balaj P. O.; Scuderi D.; Maitre P.; Ohanessian G. Vibrational signatures of protonated, phosphorylated amino acids in the gas phase. J. Am. Chem. Soc. 2008, 130, 3359–3370. 10.1021/ja073868z. 18293967

[ref43] Stedwell C. N.; Patrick A. L.; Gulyuz K.; Polfer N. C. Screening for phosphorylated and nonphosphorylated peptides by infrared photodissociation spectroscopy. Anal. Chem. 2012, 84, 9907–9912. 10.1021/ac3023058. 23078040

[ref44] Flora J. W.; Muddiman D. C. Gas-phase ion unimolecular dissociation for rapid phosphopeptide mapping by IRMPD in a penning ion trap: An energetically favored process. J. Am. Chem. Soc. 2002, 124, 6546–6547. 10.1021/ja0261170. 12047170

[ref45] Flora J. W.; Muddiman D. C. Determination of the relative energies of activation for the dissociation of aromatic versus aliphatic phosphopeptides by ESI-FTICR-MS and IRMPD. JASMS 2004, 15, 121–127. 10.1016/j.jasms.2003.10.004. 14698562

[ref46] Crowe M. C.; Brodbelt J. S. Infrared multiphoton dissociation (IRMPD) and collisionally activated dissociationof peptides in a quadrupole ion trapwith selective IRMPD of phosphopeptides. JASMS 2004, 15, 1581–1592. 10.1016/j.jasms.2004.07.016. 15519225

[ref47] Crowe M. C.; Brodbelt J. S. Differentiation of phosphorylated and unphosphorylated peptides by high-performance liquid chromatography-electrospray ionization-infrared multiphoton dissociation in a quadrupole ion trap. Anal. Chem. 2005, 77, 5726–5734. 10.1021/ac0509410. 16131088

[ref48] Ly T.; Julian R. R. Ultraviolet photodissociation: developments towards applications for mass-spectrometry-based proteomics. Angew. Chem., Int. Ed. 2009, 48, 7130–7137. 10.1002/anie.200900613. 19610000

[ref49] Madsen J. A.; Kaoud T. S.; Dalby K. N.; Brodbelt J. S. 193-nm photodissociation of singly and multiply charged peptide anions for acidic proteome characterization. Proteomics 2011, 11, 1329–1334. 10.1002/pmic.201000565. 21365762 PMC3108056

[ref50] Shaw J. B.; Li W.; Holden D. D.; Zhang Y.; Griep-Raming J.; Fellers R. T.; Early B. P.; Thomas P. M.; Kelleher N. L.; Brodbelt J. S. Complete protein characterization using top-down mass spectrometry and ultraviolet photodissociation. J. Am. Chem. Soc. 2013, 135, 12646–12651. 10.1021/ja4029654. 23697802 PMC3757099

[ref51] Brodbelt J. S. Photodissociation mass spectrometry: new tools for characterization of biological molecules. Chem. Soc. Rev. 2014, 43, 2757–2783. 10.1039/C3CS60444F. 24481009 PMC3966968

[ref52] Modzel M.; Wollenberg D. T. W.; Trelle M. B.; Larsen M. R.; Jørgensen T. J. Ultraviolet Photodissociation of Protonated Peptides and Proteins Can Proceed with H/D Scrambling. Anal. Chem. 2021, 93, 691–696. 10.1021/acs.analchem.0c02957. 33295747

[ref53] Fort K. L.; Dyachenko A.; Potel C. M.; Corradini E.; Marino F.; Barendregt A.; Makarov A. A.; Scheltema R. A.; Heck A. J. Implementation of ultraviolet photodissociation on a benchtop Q exactive mass spectrometer and its application to phosphoproteomics. Anal. Chem. 2016, 88, 2303–2310. 10.1021/acs.analchem.5b04162. 26760441

[ref54] Tsybin Y. O.; Witt M.; Baykut G.; Kjeldsen F.; Håkansson P. Combined infrared multiphoton dissociation and electron capture dissociation with a hollow electron beam in Fourier transform ion cyclotron resonance mass spectrometry. Rapid Commun. Mass Spectrom. 2003, 17, 1759–1768. 10.1002/rcm.1118. 12872281

[ref55] Caravatti P.; Allemann M. The ‘infinity cell’: A new trapped-ion cell with radiofrequency covered trapping electrodes for Fourier transform ion cyclotron resonance mass spectrometry. Org. Mass Spectrom. 1991, 26, 514–518. 10.1002/oms.1210260527.

[ref56] Wilm M.; Mann M. Analytical properties of the nanoelectrospray ion source. Anal. Chem. 1996, 68, 1–8. 10.1021/ac9509519. 8779426

[ref57] Gu C.; Tsaprailis G.; Breci L.; Wysocki V. H. Selective gas-phase cleavage at the peptide bond C-terminal to aspartic acid in fixed-charge derivatives of Asp-containing peptides. Anal. Chem. 2000, 72, 5804–5813. 10.1021/ac000555c. 11128940

[ref58] Paizs B.; Suhai S. Fragmentation pathways of protonated peptides. Mass Spectrom. Rev. 2005, 24, 508–548. 10.1002/mas.20024. 15389847

[ref59] Qin J.; Chait B. T. Preferential fragmentation of protonated gas-phase peptide ions adjacent to acidic amino acid residues. J. Am. Chem. Soc. 1995, 117, 5411–5412. 10.1021/ja00124a045.

[ref60] Schwartz B. L.; Bursey M. M. Some proline substituent effects in the tandem mass spectrum of protonated pentaalanine. Biol. Mass Spectrom. 1992, 21, 92–96. 10.1002/bms.1200210206. 1606186

[ref61] Cooper H. J.; Hudgins R. R.; Håkansson K.; Marshall A. G. Secondary fragmentation of linear peptides in electron capture dissociation. Int. J. Mass spectrom. 2003, 228, 723–728. 10.1016/S1387-3806(03)00202-1.

[ref62] Lin C.; Cournoyer J. J.; O’Connor P. B. Probing the gas-phase folding kinetics of peptide ions by IR activated DR-ECD. JASMS 2008, 19, 780–789. 10.1016/j.jasms.2008.01.001. PMC3117249 18400512

[ref63] Yang J.; Håkansson K. Characterization and optimization of electron detachment dissociation Fourier transform ion cyclotron resonance mass spectrometry. Int. J. Mass spectrom. 2008, 276, 144–148. 10.1016/j.ijms.2008.05.036.

