# Supplementary material for: Multimodal Tandem Mass Spectrometry Techniques for the Analysis of Phosphopeptides
Source: J Am Soc Mass Spectrom. 2022 May 23;33(7):1126–33. doi: 10.1021/jasms.1c00353 (PMC9264387; doi:10.1021/jasms.1c00353)
Supplement: Supplementary file 1 — js1c00353_si_001.pdf [file js1c00353_si_001.pdf]

SUPPLEMENTARY INFORMATION: MULTIMODAL TANDEM MASS SPECTROMETRY TECHNIQUES FOR THE ANALYSIS OF PHOSPHOPEPTIDES

Johanna Paris†, Alina Theisen†, Bryan P. Marzullo†, Anisha Haris†, Tomos E. Morgan†, Mark P. Barrow†, John O'Hara‡, Peter B. O'Connor†*

†University of Warwick, Department of Chemistry, Coventry, CV4 7AL, United Kingdom

‡UCB, 216 Bath Road, Slough, SL1 3WE, United Kingdom

*Corresponding author email address: p.oconnor@warwick.ac.uk

TABLE OF CONTENTS

Table S1: Peaks assignment for RDSLGpTYSSR CAD and IRMPD spectra 2

Table S2: Peaks assignment for RDSLGpTYSSR UVPD spectrum 4

Table S3: Peaks assignment for RDSLGpTYSSR ECD spectrum 5

Table S4: Peaks assignment for EVQAEQPSSpSSPR CAD spectrum 7

Table S5: Peaks assignment for EVQAEQPSSpSSPR IRMPD spectrum 8

Table S6: Peaks assignment for EVQAEQPSSpSSPR ECD spectrum 9

Table S7: Peaks assignment for EVQAEQPSSpSSPR UVPD spectrum 11

Table S8: Peaks assignment for VIEDNEpYTAR CAD spectrum 12

Table S9: Peaks assignment for VIEDNEpYTAR IRMPD spectrum 14

Table S10: Peaks assignment for VIEDNEpYTAR ECD spectrum 14

Table S11: Peaks assignment for VIEDNEpYTAR UVPD spectrum 16

Average of absolute errors is calculated as the average of the absolute ppm errors of all the assigned peaks. Standard deviation is calculated as the standard deviation of the ppm errors of all the assigned peaks.

RDSLGpTYSSR		CAD (14 V)			IRMPD (0.2 s)		
**Species**	**Theoretical *m/z***	**Observed *m/z***	**Intensity**	**ppm**	**Observed *m/z***	**Intensity**	**ppm**
$[M+2H]^{2+}$	611.26662	611.26582	1.7E+10	-1.303	611.26622	6.4E+09	-0.649
$[M+2H-H_20]^{2+}$	602.26133	602.26091	1.1E+09	-0.705	602.26119	5.6E+08	-0.240
$[M+2H-HP0_3]^{2+}$	571.28345	571.28342	3.7E+07	-0.055	*	*	*
$[M+2H-H_3PO_4]^{2+}$	562.27817	562.27782	4.7E+09	-0.621	562.27804	5.4E+09	-0.229
b2	272.13533	272.13533	6.5E+07	-0.002	272.13533	7.0E+08	-0.002
b3	359.16736	359.16736	8.4E+06	0.003	359.16741	8.4E+06	0.142
b4	472.25142	472.25144	1.6E+07	0.036	472.25158	1.5E+07	0.333
b5	529.27289	529.27295	8.3E+06	0.120	529.27324	7.4E+06	0.668
y4	512.24634	512.24626	4.4E+08	-0.151	512.24633	4.7E+08	-0.015
y3	349.18301	349.18301	4.1E+07	0.003	349.18304	1.4E+08	0.089
y2	262.15098	262.15099	1.3E+07	0.036	262.15100	8.3E+07	0.074
y1	175.11895	175.11895	1.7E+06	-0.012	175.11895	6.2E+07	-0.012
pb6	710.28690	710.28686	7.6E+06	-0.051	*	*	*
pb7	873.35022	873.35043	1.5E+07	0.235	*	*	*
pb8	960.38225	960.38226	5.3E+06	0.007	*	*	*
py8	950.39790	950.39724	6.8E+07	-0.698	950.39792	3.0E+07	0.018
py7	863.36587	863.36609	4.8E+06	0.250	*	*	*
py6	750.28181	750.28190	9.8E+06	0.119	750.28154	2.4E+06	-0.361
py5	693.26035	693.26035	2.0E+07	0.005	*	*	*
a2	244.14042	*	*	*	244.14044	1.4E+07	0.099
a3	331.17244	*	*	*	331.17252	1.4E+07	0.229
b7'	775.37333	775.37280	2.8E+07	-0.682	775.37267	4.2E+07	-0.850
b8'	862.40536	862.40541	4.0E+06	0.061	*	*	*
b9'	949.43739	949.43673	4.8E+06	-0.691	*	*	*
y9'	967.44795	967.44704	4.9E+06	-0.941	*	*	*
y8'	852.42101	852.42042	2.4E+07	-0.689	852.42040	2.5E+07	-0.713
y7'	765.38898	765.38922	2.0E+06	0.315	765.38793	6.0E+06	-1.371
y6'	652.30492	652.30456	7.9E+06	-0.544	652.30454	8.3E+06	-0.575
y5'	595.28345	595.28322	6.0E+06	-0.389	595.28373	6.5E+06	0.468
y4'	494.23577	494.23585	6.0E+06	0.156	494.23588	2.4E+07	0.217
y3'	331.17244	331.17246	3.9E+06	0.047	331.17252	1.4E+07	0.229
$C_{19}H_{11}N_5O_4^+$	373.08056	373.08053	1.5E+08	-0.068	*	*	*
$C_{19}H_9N_5O_3^+$	355.06999	355.06993	7.7E+08	-0.171	355.06993	1.8E+09	-0.171
**Average of absolute errors (ppm)**		0.296			0.337		
**Standard deviation (ppm)**		0.334			0.329		

Table S1: Peaks assignment for RDSLGpTYSSR CAD and IRMPD spectra. *: not observed. ': loss of water. '': loss of two waters. p: fragment with phosphate.

RDSLGpTYSSR		UVPD (5 shots 5 mJ)		
**Species**	**Theoretical *m/z***	**Observed *m/z***	**Intensity**	**ppm**
$[M+2H]^{2+}$	611.26662	611.26632	1.9E+10	-0.485
$[M+2H-H_20]^{2+}$	602.26133	602.26134	4.5E+08	0.009
$[M+2H-HP0_3]^{2+}$	571.28345	571.28352	1.2E+07	0.120
$[M+2H-H_3PO_4]^{2+}$	562.27817	562.27817	1.0E+09	0.002
b1	157.10839	157.10839	8.8E+07	0.016
b2	272.13533	272.13533	5.8E+08	-0.002
b3	359.16736	359.16736	2.9E+07	0.003
b4	472.25142	472.25145	2.4E+07	0.057
b5	529.27289	529.27297	2.0E+07	0.158
b9	967.44795	967.44790	1.1E+07	-0.052
y4	512.24634	512.24638	1.5E+08	0.083
y3	349.18301	349.18302	9.1E+07	0.032
y2	262.15098	262.15098	1.1E+08	-0.002
y1	175.11895	175.11895	2.2E+08	-0.012
pb6	710.28690	710.28663	3.2E+06	-0.374
pb7	873.35022	873.35041	5.6E+06	0.212
py8	950.39790	950.39790	4.4E+07	-0.003
c1	174.13494	174.13494	1.6E+07	0.020
c2	289.16188	289.16188	5.7E+07	0.001
c3	376.19391	376.19393	1.4E+07	0.058
c4	489.27797	489.27803	1.2E+07	0.119
c5	546.29944	546.29944	1.1E+07	0.008
c2.	288.15405	288.15406	2.8E+06	0.019
c3.	375.18608	375.18610	4.4E+06	0.045
z4	496.22761	496.22777	4.2E+06	0.316
z3	333.16428	333.16429	6.0E+07	0.015
z2	246.13226	246.13225	2.3E+07	-0.026
z1	159.10023	159.10024	1.6E+07	0.075
z4.	497.23544	497.23542	4.5E+06	-0.037
z3.	334.17211	334.17213	5.0E+06	0.060
pc6	727.31345	727.31353	4.4E+06	0.117
a2	244.14042	244.14041	8.5E+07	-0.024
a3	331.17244	331.17244	4.7E+07	-0.013
a4	444.25651	444.25654	2.5E+07	0.071
a7	765.38898	765.38904	1.0E+07	0.080
a8	852.42101	852.42105	1.4E+08	0.050
x4	538.22560	538.22574	5.5E+06	0.256
x3	375.16227	375.16231	7.0E+06	0.097
x2	288.13025	288.13025	7.7E+06	0.017
x1	201.09822	201.09822	1.3E+07	0.016
pa6	682.29198	682.29215	3.5E+06	0.247
pa7	845.35531	845.35539	1.8E+07	0.095
pa8	932.38734	932.38803	6.8E+06	0.742

b7'	775.37333	775.37327	2.6E+07	-0.076
a7'	747.37841	747.37838	1.2E+07	-0.046
b8'	862.40536	862.40541	5.0E+06	0.061
b9'	949.43739	949.43724	1.2E+07	-0.153
y9'	967.44795	967.44790	1.1E+07	-0.052
y8'	852.42101	852.42105	1.4E+08	0.050
y7'	765.38898	765.38904	1.0E+07	0.080
y6'	652.30492	652.30482	1.4E+07	-0.146
y5'	595.28345	595.28353	2.3E+07	0.132
y4'	494.23577	494.23582	2.0E+07	0.095
y3'	331.17244	331.17240	4.3E+06	-0.134
y2'	244.14042	244.14041	8.5E+07	-0.024
$C_{19}H_9N_5O_3^+$	355.06999	355.06993	4.6E+06	-0.171
**Average of absolute errors (ppm)**		0.098		
**Standard deviation (ppm)**		0.129		

Table S2: Peaks assignment for RDSLGpTYSSR UVPD spectrum. ': loss of water. p: fragment with phosphate.

RDSLGpTYSSR		ECD (0.2 s 1.2 V)		
**Species**	**Theoretical *m/z***	**Observed *m/z***	**Intensity**	**ppm**
$[M+2H]^{2+}$	611.26662	611.26674	2.4E+09	0.202
b2	272.13533	272.13532	2.9E+06	-0.039
y3	349.18301	349.18302	1.4E+07	0.032
y2	262.15098	262.15099	5.0E+06	0.036
c1	174.13494	174.13494	2.4E+07	0.020
c2	289.16188	289.16188	2.9E+07	0.001
c3	376.19391	376.19392	6.2E+07	0.032
c4	489.27797	489.27796	5.0E+06	-0.024
c5	546.29944	546.29944	4.5E+07	0.008
c1.	173.12711	173.12711	1.2E+07	-0.009
c2.	288.15405	288.15406	4.5E+07	0.019
c3.	375.18608	375.18608	1.1E+08	-0.008
c4.	488.27015	488.27015	2.4E+07	0.006
c5.	545.29161	545.29161	8.5E+07	-0.001
z4	496.22761	496.22757	1.6E+07	-0.088
z3	333.16428	333.16427	8.1E+06	-0.045
z4.	497.23544	497.23543	1.3E+08	-0.017
z3.	334.17211	334.17211	1.2E+08	0.000
z2.	247.14008	247.14008	3.4E+07	-0.006
pc6	727.31345	727.31317	4.4E+06	-0.378
pc9	1064.44083	1064.44142	5.5E+07	0.554
pc7.	889.36895	889.36893	3.1E+07	-0.021
pc8.	976.40098	976.40106	2.0E+07	0.085
pc9.	1063.43301	1063.43303	3.3E+07	0.023
pz8.	935.38700	935.38687	7.8E+06	-0.143
pz7.	848.35498	848.35458	2.5E+06	-0.466
**Average of absolute errors (ppm)**		0.087		
**Standard deviation (ppm)**		0.146		

Table S3: Peaks assignment for RDSLGpTYSSR ECD spectrum. p: fragment with phosphate.

EVQAEQPSSpSSPR	CAD (14 V)			
**Species**	**Theoretical *m/z***	**Observed *m/z***	**Intensity**	**ppm**
$[M+2H]^{2+}$	741.31703	741.31633	2.1E+10	-0.951
$[M+2H-H_20]^{2+}$	732.31175	732.31167	2.1E+09	-0.113
$[M+2H-H_3PO_4]^{2+}$	692.32859	692.32849	9.9E+08	-0.140
b2	229.11828	229.11828	2.5E+06	-0.015
b3	357.17686	357.17683	1.7E+08	-0.087
b4	428.21397	428.21397	1.5E+08	-0.011
b5	557.25657	557.25644	1.3E+09	-0.229
b6	685.31515	685.31496	1.5E+09	-0.270
b7	782.36791	782.36759	3.0E+07	-0.408
b8	869.39994	869.39981	6.6E+07	-0.147
b9	956.43197	956.43209	6.1E+07	0.130
y4	446.23577	446.23577	3.4E+06	-0.006
y3	359.20374	359.20369	1.4E+08	-0.151
y2	272.17172	272.17167	7.4E+07	-0.169
pb10	1123.43033	1123.43030	1.5E+07	-0.022
pb11	1210.46235	1210.46293	2.0E+07	0.476
py10	1125.45721	1125.45715	5.9E+08	-0.052
py9	1054.42010	1054.41977	1.0E+08	-0.308
py8	925.37750	925.37755	7.4E+08	0.052
py7	797.31892	797.31821	8.6E+09	-0.896
py6	700.26616	700.26615	1.8E+08	-0.015
py5	613.23413	613.23413	1.5E+08	-0.003
py4	526.20210	526.20207	1.9E+08	-0.064
a4	400.21906	400.21903	7.5E+06	-0.075
a5	529.26165	529.26152	3.7E+07	-0.252
a6	657.32023	657.32017	9.1E+06	-0.092
y10'	1027.48031	1027.48020	1.9E+07	-0.110
y8'	827.40061	827.40053	3.9E+07	-0.092
y7'	699.34203	699.34173	3.3E+08	-0.427
y6'	602.28926	602.28913	1.1E+07	-0.224
y5'	515.25724	515.25711	4.3E+07	-0.246
y4'	428.22521	428.22519	4.2E+07	-0.042
y10''	1009.46975	1009.46954	1.1E+07	-0.207
y9''	938.43263	938.43277	2.4E+07	0.144
y8''	809.39004	809.38958	8.5E+07	-0.570
y7''	681.33146	681.33138	2.1E+08	-0.123
y6''	584.27870	584.27872	6.2E+06	0.034
y5''	497.24667	497.24657	1.3E+08	-0.205
b2'	211.10772	211.10773	6.0E+06	0.053
b3'	339.16630	339.16620	4.7E+08	-0.284
b4'	410.20341	410.20330	6.9E+08	-0.268
b5'	539.24600	539.24579	2.3E+09	-0.395
b6'	667.30458	667.30442	8.7E+08	-0.241

b7'	764.35734	764.35715	2.5E+07	-0.254
b8'	851.38937	851.38927	4.6E+07	-0.121
b9'	938.42140	938.42146	2.6E+07	0.063
b10'	1025.45343	1025.45380	2.6E+07	0.361
b11'	1112.48546	1112.48491	4.4E+07	-0.493
py11^{2+}	627.26153	627.26144	9.3E+08	-0.146
py11^{2+}'	618.25625	618.25618	1.3E+08	-0.112
py9'	1036.40953	1036.40964	2.8E+08	0.106
py8'	907.36694	907.36668	2.3E+09	-0.283
py7'	779.30836	779.30790	1.7E+08	-0.590
**Average of absolute errors (ppm)**		0.209		
**Standard deviation (ppm)**		0.205		

Table S4: Peaks assignment for EVQAEQPSSpSSPR CAD spectrum. ': loss of water. '': loss of two waters. p: fragment with phosphate.

EVQAEQPSSpSSPR		IRMPD (0.2 s)		
**Species**	**Theoretical *m/z***	**Observed *m/z***	**Intensity**	**ppm**
$[M+2H]^{2+}$	741.31703	741.31663	3.4E+09	-0.546
$[M+2H-H_20]^{2+}$	732.31175	732.31195	6.9E+07	0.270
$[M+2H-H_3PO_4]^{2+}$	692.32859	692.32854	5.8E+07	-0.068
b2	229.11828	229.11825	5.2E+06	-0.146
b3	357.17686	357.17680	3.7E+07	-0.171
b4	428.21397	428.21397	1.2E+07	-0.011
b5	557.25657	557.25657	1.6E+08	0.004
b6	685.31515	685.31520	6.1E+07	0.080
b8	869.39994	869.39934	7.5E+06	-0.687
b9	956.43197	956.43272	8.9E+06	0.788
y3	359.20374	359.20369	1.1E+08	-0.151
y2	272.17172	272.17165	2.0E+06	-0.242
y1	175.11895	175.11895	1.1E+07	-0.013
py10	1125.45721	1125.45720	9.3E+07	-0.008
py8	925.37750	925.37751	1.6E+07	0.009
py7	797.31892	797.31900	9.6E+08	0.095
py6	700.26616	700.26646	2.7E+07	0.428
py5	613.23413	613.23412	1.4E+07	-0.020
py4	526.20210	526.20214	3.5E+07	0.069
y7'	699.34203	699.34214	7.2E+07	0.159
y6'	602.28926	602.28932	2.8E+06	0.091
y5'	515.25724	515.25730	1.7E+07	0.123
y4'	428.22521	428.22520	3.6E+07	-0.019
y3'	341.19318	341.19318	1.5E+06	0.001
y8''	809.39004	809.38999	2.9E+07	-0.064
y7''	681.33146	681.33162	5.9E+07	0.229
y5''	497.24667	497.24670	6.7E+07	0.057
b2'	211.10772	211.10770	1.9E+07	-0.089
b3'	339.16630	339.16622	3.0E+08	-0.225
b4'	410.20341	410.20332	4.2E+08	-0.220
b5'	539.24600	539.24589	6.5E+08	-0.210
b6'	667.30458	667.30459	7.8E+07	0.014
b7'	764.35734	764.35741	4.2E+06	0.086
b11'	1112.48546	1112.48547	4.0E+06	0.011
$py11^{2+}$	627.26153	627.26138	6.1E+07	-0.241
$py11^{2+\prime}$	618.25625	618.25625	7.6E+07	0.002
py9'	1036.40953	1036.40973	6.3E+07	0.193
py8'	907.36694	907.36693	5.7E+08	-0.008
py7'	779.30836	779.30838	5.5E+07	0.026
**Average of absolute errors (ppm)**		0.161		
**Standard deviation (ppm)**		0.190		

Table S5: Peaks assignment for EVQAEQPSSpSSPR IRMPD spectrum. ': loss of water. '': loss of two waters. p: fragment with phosphate.

EVQAEQPSSpSSPR		ECD (0.2 s 1.2 V)		
**Species**	**Theoretical *m/z***	**Observed *m/z***	**Intensity**	**ppm**
$[M+2H]^{2+}$	741.31703	741.31657	1.3E+10	-0.627
$[M+2H-H_20]^{2+}$	732.31175	732.31212	1.3E+07	0.502
y3	359.20374	359.20379	2.0E+06	0.127
py11	1253.51579	1253.51657	3.3E+07	0.625
py10	1125.45721	1125.45717	2.2E+07	-0.035
py8	925.37750	925.37780	1.2E+07	0.322
py7	797.31892	797.31897	1.7E+07	0.057
z3	343.18502	343.18501	1.1E+07	-0.030
z3.	344.19285	344.19285	1.2E+08	0.013
pz12	1336.56548	1336.56585	7.7E+07	0.280
pz11	1237.49706	1237.49708	5.1E+07	0.015
pz10	1109.43849	1109.43854	4.3E+07	0.050
pz9	1038.40137	1038.40136	1.4E+08	-0.011
pz8	909.35878	909.35876	1.1E+08	-0.020
pz4	510.18338	510.18352	4.2E+07	0.275
pz8.	910.36660	910.36673	1.9E+08	0.140
pz6.	685.25526	685.25512	2.3E+07	-0.206
pz5.	598.22323	598.22324	1.7E+07	0.012
pz4.	511.19120	511.19118	4.4E+07	-0.048
z12'	1238.58858	1238.58887	5.1E+07	0.234
z11'	1139.52017	1139.52038	6.2E+07	0.187
z10'	1011.46159	1011.46148	1.7E+08	-0.108
z9'	940.42448	940.42476	7.8E+06	0.303
z8'	811.38188	811.38183	3.5E+07	-0.064
pz8'	891.34821	891.34807	2.0E+07	-0.161
pw13	1394.57096	1394.57115	9.6E+07	0.140
pw12	1265.52836	1265.52820	4.0E+07	-0.128
pw11	1166.45995	1166.46052	2.4E+07	0.490
pw9	967.36426	967.36435	1.9E+07	0.096
**Average of absolute errors (ppm)**		0.183		
**Standard deviation (ppm)**		0.178		

Table S6: Peaks assignment for EVQAEQPSSpSSPR ECD spectrum. ': loss of water. p: fragment with phosphate. w: z-C_3H_5NO

EVQAEQPSSpSSPR		UVPD (5 shots 5 mJ)		
**Species**	**Theoretical *m/z***	**Observed *m/z***	**Intensity**	**ppm**
$[M+2H]^{2+}$	741.31703	741.31638	7.1E+09	-0.088
$[M+2H-H_2O]^{2+}$	732.31175	732.31162	2.5E+08	-0.018
$[M+2H-H_3PO_4]^{2+}$	692.32859	692.32849	2.5E+07	-0.014
$[M+2H-CO]^{2+}$	727.31958	727.31949	2.8E+07	-0.012
a1	102.05495	102.05495	4.0E+06	-0.005
b1	130.04987	130.04987	2.3E+06	0.000
c1	147.07642	147.07642	5.2E+06	0.001
y1'	157.10839	157.10839	5.7E+06	0.002
z1	159.10023	159.10022	5.9E+06	-0.005
y1	175.11895	175.11895	4.1E+07	-0.001
x1	201.09822	201.09822	6.0E+06	0.002
a2	201.12337	201.12337	1.6E+07	0.001
b2'	211.10772	211.10772	7.0E+07	0.001
b2	229.11828	229.11829	1.1E+07	0.003
y2'	254.16115	254.16115	8.1E+06	-0.001
y2	272.17172	272.17171	1.0E+08	-0.002
y3''	323.18262	323.18256	4.4E+06	-0.017
a3	329.18195	329.18197	1.1E+08	0.007
b3'	339.16630	339.16629	2.4E+08	-0.002
y3'	341.19318	341.19319	5.1E+06	0.003
z3	343.18502	343.18504	6.0E+06	0.006
b3	357.17686	357.17685	2.9E+07	-0.003
y3	359.20374	359.20375	5.8E+07	0.002
b4'-CO	382.20850	382.20851	2.1E+07	0.004
x3	385.18301	385.18298	2.9E+06	-0.008
a4	400.21906	400.21904	1.9E+06	-0.005
b4'	410.20341	410.20341	1.9E+08	0.000
y4''	410.21464	410.21461	6.5E+06	-0.008
b4	428.21397	428.21401	1.0E+07	0.008
y4'	428.22521	428.22522	3.8E+07	0.003
z4.	431.22487	431.22482	2.7E+06	-0.012
y5''	497.24667	497.24669	5.8E+07	0.004
y5'	515.25724	515.25725	1.9E+07	0.003
b5'-CO	511.25109	511.25117	7.2E+06	0.016
y6''	584.27870	584.27869	4.4E+06	-0.002
b5''	521.23544	521.23544	1.7E+07	0.000
a5	529.26165	529.26173	4.1E+06	0.015
b5'	539.24600	539.24601	1.1E+08	0.001
b5	557.25657	557.25658	5.1E+07	0.002
y6'	602.28926	602.28931	1.7E+06	0.007
c5	574.28312	574.28309	1.3E+07	-0.005
y7'''	663.32090	663.32102	9.8E+06	0.018
y7''	681.33146	681.33146	5.0E+07	-0.001

y7'	699.34203	699.34204	1.1E+08	0.002
y8'''	791.37948	791.37928	2.9E+07	-0.025
y8''	809.39004	809.39002	5.0E+07	-0.003
b6'	667.30458	667.30464	3.2E+07	0.009
y9'''	920.42207	920.42242	7.4E+06	0.038
y9''	938.43263	938.43268	1.8E+07	0.005
b6	685.31515	685.31519	1.9E+07	0.007
y10''	1009.46975	1009.46944	6.2E+06	-0.031
y10'	1027.48031	1027.48032	1.9E+07	0.001
$py8^{2+}$	463.19239	463.19228	1.8E+06	-0.024
py4	526.20210	526.20213	1.9E+07	0.005
a7	754.37299	754.37371	1.9E+06	0.095
$py10^{2+}$	563.23224	563.23203	1.9E+06	-0.038
b7'	764.35734	764.35728	2.0E+06	-0.008
py5	613.23413	613.23406	5.8E+06	-0.012
$py11^{2+}$	627.26153	627.26159	5.2E+06	0.009
pz6	684.24744	684.24740	3.2E+06	-0.005
py6	700.26616	700.26624	2.0E+07	0.011
b8'	851.38937	851.39008	2.5E+06	0.083
b8	869.39994	869.39893	2.7E+06	-0.116
py7''	761.29780	761.29774	1.4E+07	-0.007
py7'	779.30836	779.30838	8.3E+07	0.003
py7	797.31892	797.31896	1.8E+08	0.004
pz8-H-H_2O	890.34039	890.34064	4.6E+06	0.028
pz8-H	908.35095	908.35107	4.7E+07	0.013
pz8	909.35878	909.35437	1.9E+07	-0.485
pz8.	910.36660	910.35763	4.3E+06	-0.986
py8	925.37750	925.37764	2.5E+07	0.015
py10	1125.45721	1125.45711	1.7E+07	-0.009
pc10	1140.45687	1140.45661	2.9E+06	-0.023
b3-C_2H_6O	311.13500	311.13499	1.8E+07	-0.002
**Average of absolute errors (ppm)**		0.033		
**Standard deviation (ppm)**		0.126		

Table S7: Peaks assignment for EVQAEQPSSpSSPR UVPD spectrum. ': loss of water. '': loss of two water. p: fragment with phosphate.

VIEDNEpYTAR		CAD (14 V)		
**Species**	**Theoretical *m/z***	**Observed *m/z***	**Intensity**	**ppm**
$[M+2H]^{2+}$	645.27411	645.27390	4.2E+09	-0.321
[M+2H]'	636.26883	636.26871	4.7E+08	-0.181
[M+2H]''	627.26354	627.26336	5.3E+07	-0.291
a2	185.16484	185.16484	6.7E+07	0.001
a3	314.20743	314.20752	3.2E+06	0.277
a5	543.27730	543.27732	2.1E+06	0.031
b2	213.15975	213.15977	1.2E+08	0.073
b3	342.20235	342.20238	7.7E+07	0.095
b4	457.22929	457.22932	7.4E+07	0.065
b5	571.27222	571.27220	3.6E+07	-0.031
b5'	553.26165	553.26175	2.7E+07	0.175
b6	700.31481	700.31471	4.3E+07	-0.144
b6'	682.30425	682.30412	8.1E+06	-0.185
pb7	943.34447	943.34398	3.3E+08	-0.520
pb7'	925.33391	925.33349	1.6E+07	-0.449
pb8	1044.39215	1044.39212	5.0E+06	-0.031
pb9	1115.42926	1115.42939	1.2E+07	0.114
py4	590.23340	590.23335	2.8E+08	-0.091
py5	719.27600	719.27574	1.3E+08	-0.357
py5'	701.26543	701.26523	5.0E+07	-0.288
py6	833.31892	833.31873	2.2E+08	-0.233
py6'	815.30836	815.30839	1.9E+07	0.037
$py6^{2+}$	417.16310	417.16316	5.9E+06	0.143
$py6^{2+'}$	408.15782	408.15774	2.6E+06	-0.191
$py6^{2+''}$	399.15254	399.15255	3.9E+06	0.036
py7	948.34587	948.34575	1.4E+08	-0.124
$py7^{2+}$	474.67657	474.67654	2.2E+06	-0.067
py8	1077.38846	1077.38798	3.5E+08	-0.446
py8'	1059.37790	1059.37744	7.2E+08	-0.430
$py8^{2+}$	539.19787	539.19784	3.8E+08	-0.053
$py8^{2+'}$	530.19259	530.19256	1.2E+08	-0.049
$py8^{2+''}$	521.18730	521.18725	9.1E+07	-0.103
$py8^{2+'''}$	512.18202	512.18191	4.1E+07	-0.218
py9	1190.47252	1190.47173	3.6E+06	-0.667
$py9^{2+'}$	586.73462	586.73464	1.5E+07	0.037
$py9^{2+}$	595.73990	595.73980	1.0E+08	-0.169
y1	175.11895	175.11896	7.6E+06	0.045
y2	246.15607	246.15609	1.6E+07	0.098
y3	347.20374	347.20377	8.4E+07	0.074
**Average of absolute errors (ppm)**		0.174		
**Standard deviation (ppm)**		0.156		

Table S8: Peaks assignment for VIEDNEpYTAR CAD spectrum. ': loss of water. '': loss of two water. ''': loss of three water. p: fragment with phosphate.

VIEDNEpYTAR		IRMPD (0.15 s)		
**Species**	**Theoretical *m/z***	**Observed *m/z***	**Intensity**	**ppm**
$[M+2H]^{2+}$	645.27411	645.27384	7.2E+09	-0.414
$[M+2H]^{2+\prime}$	636.26883	636.26868	8.5E+08	-0.228
$[M+2H]^{2+\prime\prime}$	627.26354	627.26347	8.8E+07	-0.116
a2	185.16484	185.16484	1.2E+08	0.001
a4'	411.22381	411.22367	2.1E+06	-0.343
b2	213.15975	213.15975	5.2E+08	-0.020
b3	342.20235	342.20235	7.8E+07	0.007
b3'	324.19178	324.19177	6.5E+06	-0.039
b4	457.22929	457.22929	1.3E+08	-0.001
b4'	439.21873	439.21873	1.4E+07	0.010
$b4^{2+}$	229.11828	229.11831	1.6E+06	0.116
b5	571.27222	571.27224	3.7E+07	0.039
b5'	553.26165	553.26161	2.6E+07	-0.078
b6	700.31481	700.31482	1.9E+07	0.013
b6'	682.30425	682.30431	3.0E+07	0.093
b6''	664.29368	664.29352	2.1E+07	-0.243
pb7	943.34447	943.34428	1.9E+07	-0.202
pb8	1044.39215	1044.39177	3.0E+06	-0.363
pb9	1115.42926	1115.42927	2.2E+07	0.007
py4	590.23340	590.23339	2.3E+08	-0.023
py4'	572.22284	572.22278	2.1E+07	-0.103
py5	719.27600	719.27599	1.5E+07	-0.010
py5'	701.26543	701.26541	6.3E+07	-0.032
py6	833.31892	833.31896	1.7E+08	0.043
py6'	815.30836	815.30842	9.9E+07	0.074
$py6^{2+}$	417.16310	417.16302	4.1E+06	-0.193
py7	948.34587	948.34583	1.3E+08	-0.039
py7'	930.33530	930.33518	4.4E+07	-0.132
py8	1077.38846	1077.38876	4.0E+06	0.278
py8'	1059.37790	1059.37795	3.9E+08	0.051
py8''	1041.36733	1041.36724	1.3E+08	-0.087
py8'''	1023.35677	1023.35643	2.1E+07	-0.329
$py8^{2+}$	539.19787	539.19781	1.8E+08	-0.108
$py8^{2+\prime}$	530.19259	530.19253	2.7E+07	-0.106
$py8^{2+\prime\prime}$	521.18730	521.18727	4.1E+07	-0.065
$py8^{2+\prime\prime\prime}$	512.18202	512.18204	3.7E+07	0.036
y8'	961.40100	961.40128	2.0E+07	0.291
$py9^{2+}$	595.73990	595.73982	1.2E+07	-0.135
y1	175.11895	175.11895	2.2E+08	-0.012
y2	246.15607	246.15607	8.8E+07	0.016
y2'	228.14550	228.14549	4.0E+06	-0.049
y3	347.20374	347.20374	1.9E+08	-0.013

y3'	329.19318	329.19317	1.1E+07	-0.030
y4	510.26707	510.26715	6.2E+06	0.151
y4'	492.25651	492.25656	9.0E+06	0.105
y6'	735.34203	735.34221	9.6E+06	0.246
y6''	717.33146	717.33151	4.1E+06	0.064
**Average of absolute errors (ppm)**		0.110		
**Standard deviation (ppm)**		0.109		

Table S9: Peaks assignment for VIEDNEpYTAR IRMPD spectrum. ': loss of water. '': loss of two water. ''': loss of three water. p: fragment with phosphate.

**VIEDNEpYTAR**		**ECD (0.3 s 1.2 V)**		
**Species**	**Theoretical *m/z***	**Observed *m/z***	**Intensity**	**ppm**
[M+2H]2+	645.27411	645.27406	3.3E+09	-0.073
z1.	160.10805	160.10805	1.7E+07	-0.020
z2	230.13734	230.13735	7.0E+07	0.035
z2.	231.14517	231.14518	8.2E+07	0.057
z3	331.18502	331.18503	4.3E+07	0.029
z3.	332.19285	332.19285	2.4E+08	0.014
pz4'	556.20412	556.20414	6.3E+06	0.045
pz4	574.21468	574.21468	6.8E+07	0.000
pz4.	575.22250	575.22251	1.2E+08	0.009
pz5'	685.24671	685.24668	4.7E+07	-0.041
pz5	703.25727	703.25727	1.7E+08	-0.004
pz5.	704.26510	704.26508	1.9E+08	-0.025
pz6'	799.28964	799.28954	2.3E+07	-0.120
pz6	817.30020	817.30021	3.9E+08	0.012
pz6.	818.30803	818.30867	7.7E+07	0.788
pz7-CO_2	888.33731	888.33735	9.2E+07	0.040
pz7	932.32714	932.32716	1.1E+08	0.018
pc7	960.37102	960.37129	4.2E+06	0.282
pz8-CO_2	1017.37991	1017.37992	2.2E+07	0.013
pz8	1061.36974	1061.36965	3.6E+07	-0.081
pz9	1174.45380	1174.45332	2.2E+07	-0.409
$[M+2H+e]^+$	1289.53984	1289.54041	1.6E+08	0.441
**Average of absolute errors (ppm)**		0.116		
**Standard deviation (ppm)**		0.191		

Table S10: Peaks assignment for VIEDNEpYTAR ECD spectrum. ': loss of water. p: fragment with phosphate.

VIEDNEpYTAR		UVPD (5 shots 5mJ)		
**Species**	**Theoretical *m/z***	**Observed *m/z***	**Intensity**	**ppm**
$[M+2H]^{2+}$	645.27411	645.27353	7.0E+09	-0.895
$[M+2H-H_20]^{2+}$	636.26883	636.26874	7.0E+07	-0.134
z1	159.10023	159.10023	3.4E+06	0.012
y1	175.11895	175.11895	9.0E+07	-0.012
a2	185.16484	185.16483	2.0E+08	-0.053
x1	201.09822	201.09822	2.3E+06	0.016
x1+H	202.10604	202.10605	1.5E+06	0.041
b2	213.15975	213.15975	1.4E+08	-0.020
$b4^{2+}$	229.11828	229.11828	1.8E+06	-0.015
z2	230.13734	230.13735	5.1E+06	0.035
y2-2H	244.14042	244.14039	1.0E+06	-0.106
y2	246.15607	246.15607	3.6E+07	0.016
x2	272.13533	272.13530	2.0E+06	-0.112
x2+H	273.14316	273.14315	2.0E+06	-0.020
a3	314.20743	314.20745	2.0E+06	0.055
z3	331.18502	331.18503	9.4E+06	0.029
b3	342.20235	342.20235	6.6E+06	0.007
y3-H	346.19592	346.19605	1.0E+06	0.377
y3	347.20374	347.20375	3.4E+07	0.016
x3+H	374.19083	374.19089	1.8E+06	0.150
b4'	439.21873	439.21873	1.4E+06	0.010
b4	457.22929	457.22929	4.5E+06	-0.001
y4'	492.25651	492.25656	4.9E+06	0.105
y4	510.26707	510.26706	7.6E+06	-0.025
$py8^{2+}$	539.19787	539.19778	6.4E+06	-0.164
a5+H	544.28513	544.28513	1.3E+06	0.003
pz4	574.21468	574.21466	2.6E+06	-0.034
py4	590.23340	590.23338	4.7E+07	-0.040
px4+H	617.22049	617.22042	2.9E+06	-0.119
a6+H	673.32772	673.32755	5.7E+06	-0.255
py5''	683.25487	683.25473	3.4E+06	-0.201
b6	700.31481	700.31492	2.2E+06	0.156
py5'	701.26543	701.26532	2.4E+07	-0.160
py5	719.27600	719.27573	2.2E+06	-0.371
y6'	735.34203	735.34203	4.9E+06	0.002
y6	753.35259	753.35201	2.1E+06	-0.775
py6'	815.30836	815.30765	3.6E+07	-0.870
py6	833.31892	833.31882	4.3E+07	-0.125
pa7+H	916.35738	916.35744	7.0E+06	0.065
py7'	930.33530	930.33493	1.7E+07	-0.401
pb7	943.34447	943.34450	1.8E+06	0.031
py7	948.34587	948.34561	1.2E+07	-0.271
py8''	1041.36733	1041.36661	2.5E+07	-0.692

py8'	1059.37790	1059.37732	4.2E+07	-0.544
pb9	1115.42926	1115.42926	2.4E+06	-0.002
**Average of absolute errors (ppm)**		0.152		
**Standard deviation (ppm)**		0.213		

Table S11: Peaks assignment for VIEDNEpYTAR UVPD spectrum. ': loss of water. '': loss of two water. ''': loss of three water. p: fragment with phosphate.
